# Serum bile acids and GLP-1 decrease following telemetric induced weight loss: results of a randomized controlled trial

**DOI:** 10.1038/srep30173

**Published:** 2016-07-25

**Authors:** Ronald Biemann, Marina Penner, Katrin Borucki, Sabine Westphal, Claus Luley, Raik Rönicke, Kathleen Biemann, Cornelia Weikert, Anke Lux, Nikolai Goncharenko, Hanns-Ulrich Marschall, Jochen G. Schneider, Berend Isermann

**Affiliations:** 1 Institute of Clinical Chemistry and Pathobiochemistry, Otto-von-Guericke University, Magdeburg, Germany; 2 The Federal Institute for Risk Assessment, Department of Food Safety, Berlin, Germany; 3 Institute of Social Medicine, Epidemiology, and Health Economics, Charité University Medical Center, Berlin, Germany; 4 Institute for Biometrics and Medical Informatics, Otto-von-Guericke University, Magdeburg, Germany; 5 IBBL (Integrated BioBank of Luxembourg), Luxemburg City, Luxembourg; 6 Sahlgrenska Academy, Institute of Medicine, Department of Molecular and Clinical Medicine, University of Gothenburg, Gothenburg, Sweden; 7 Luxembourg Centre for Systems Biomedicine (LCSB), University of Luxembourg; 8 Department of Internal Medicine II, Saarland University Medical Center at Homburg/Saar, Homburg, Germany

## Abstract

Bile acids (BAs) are increasingly recognised as metabolic regulators, potentially improving insulin sensitivity following bariatric surgery. However, physiological relevance of such observations remains unknown. Hence, we analysed serum BA composition and associated gut-derived hormone levels following lifestyle-induced weight loss in individuals with metabolic syndrome (MetS). 74 non-smoking men (45–55 yr) with MetS were randomised to a lifestyle-induced weight loss program (supervision *via* telemonitoring) or to a control arm. Before and after a 6 months intervention period clinical and laboratory parameters, body composition, serum BA profile, FGF-19, and GLP-1 concentrations were determined in fasting blood samples. 30 participants in the control and 33 participants in the treatment arm completed the study and were included in the data analysis. In participants of the treatment arm lifestyle-induced weight loss resulted in markedly improved insulin sensitivity. Serum levels of BA species and total GLP-1 decreased, while FGF-19 remained stable. Serum BA composition changed towards an increased 12α-hydroxylated/non-12α-hydroxylated ratio. None of these parameters changed in participants of the control arm. Our results demonstrate that improved metabolic control by lifestyle modifications lowers serum levels of BAs and GLP-1 and changes serum BA composition towards an increased 12α/non-12α ratio (ICTRP Trial Number: U1111-1158-3672).

The clustering of diabetes, raised fasting plasma glucose, abdominal obesity, high cholesterol and high blood pressure, all well-established cardiovascular risk factors, is commonly referred to as the metabolic syndrome (MetS). Its increasing prevalence represents a major public health burden as individuals with MetS have twice the risk of developing cardiovascular disease (CVD) and a 5-fold elevated risk for type 2 diabetes^1^. Moreover, the risk for CVD and type 2 diabetes increases with the number of MetS components present^2^. So far, lifestyle-induced weight loss is regarded as efficient therapy to reverse MetS and to prevent type 2 diabetes and CVD in individuals with MetS^3^,^4^.

Bile acids (BAs) are considered to be important metabolic regulators^5^. In addition to their function in absorption of lipophilic nutrients, BAs act as ligands for the nuclear farnesoid X receptor (FXR) and the G-protein-coupled BA receptor (TGR5) that control intestinal secretion of fibroblast growth factor 19 (FGF-19) and the incretin glucagon-like peptide-1 (GLP-1)^6^,^7^,^8^. FGF19 and GLP-1 regulate glucose but also BA homeostasis^8^,^9^,^10^,^11^. Hence, control of GLP-1 and FGF-19 secretion by BA receptors suggests a feedback loop between BAs and glucose homeostasis. Accordingly, it has been observed that bariatric surgery, e.g. gastric bypass surgery, which may increase serum BA, FGF-19 and GLP-1 levels, is coupled with rapid resolution of insulin resistance and improved insulin secretion^9^,^12^,^13^,^14^. Furthermore, plasma BA composition was recently suggested as early biomarker for insulin resistance in healthy non-obese individuals^15^.

However, it remains unknown whether therapeutically altering of serum BA levels and/or composition may represent a useful target for interventions aiming to improve glucose metabolism in insulin resistant individuals^9^,^10^,^16^. Of note, prospective clinical studies evaluating the orchestrated changes of serum BA composition and associated gut-derived hormone levels (e.g. GLP-1, FGF-19) during conventional (lifestyle-induced) weight loss are lacking. To address the question how conventional weight loss affects BA metabolism in prediabetic individuals we prospectively determined the changes and interrelations of 15 endogenous BA species, clinical and metabolic parameters, and the intestinal hormones GLP-1 and FGF-19 following lifestyle-induced weight loss in 74 well-defined individuals with MetS in a prospective study.

## Results

### Clinical and laboratory parameters

Thirty participants in the control and 33 participants in the telemonitored lifestyle-induced weight loss arm (Active Body Control, ABC, http://www.abcprogramm.de) completed the study (85% of the study population) and were included in the data analysis. 2 participants of the ABC arm and 4 of the control arm did not follow the study protocol, and 3 participants of the control arm left the study because not being randomised to participate in the weight loss program. The remaining two dropouts of the ABC arm declined for undisclosed reasons. The study population did not differ in the distribution of age, sex and parameters of the MetS (Table 1).

**Figure 1:**
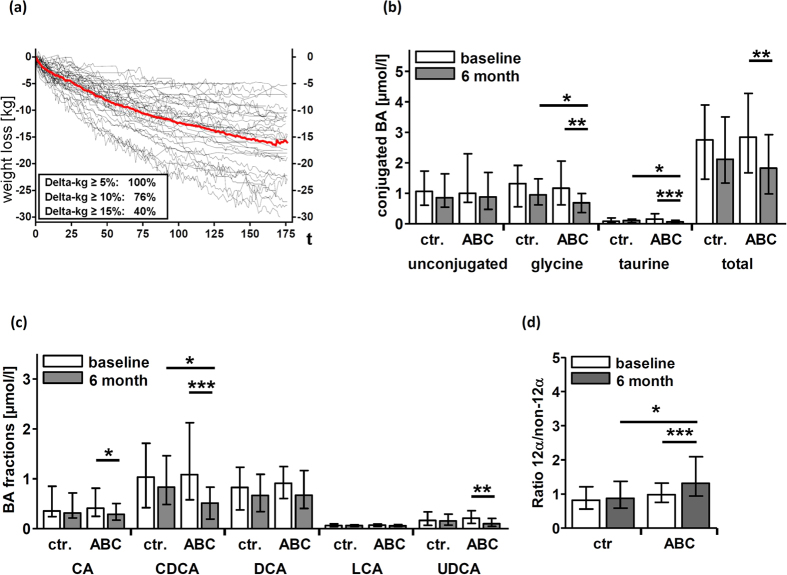
Changes of body weight and bile acid (BA) fractions in subjects with MetS who underwent lifestyle-induced weight loss (ABC) and in controls. Body weight was daily telemonitored in participants who attended the lifestyle-induced weight loss program and median weight progress is demonstrated as bold line (**a**). Concentrations of conjugated BAs (**b**) and BA fractions (**c**) at baseline and 6 months are shown as bar graphs. Ratio between 12α-hydroxylated BAs and non-12α-hydroxylated BAs (**c**). Wilcoxon Signed-Rank Test was used to analyse differences between paired samples; data are presented as median ± interquartile range; *p < 0.05; **p < 0.01; ***p < 0.01.

At baseline and after 6 months clinical parameters and body composition were determined in participants of both arms (Table 1). Participants of the ABC arm reduced their individual body weight by at least 5% (Fig. 1a). Notably, 76% reduced their body weight by at least 10% and 40% reduced their body weight by at least 15%. Absolute and relative weight reductions are given in supplementary Table S1. Reduction of body weight was mainly attributable to a significant reduction of individual body fat mass (−23,57%, p < 0.001), especially in the trunk region (−24.96%, p < 0.001). In controls, BMI and other parameters of the body composition remained unchanged and trunk fat mass even increased by 4.78%. In both groups, no progression to overt type 2 diabetes was observed.

ABC program led to significant reductions of systolic and diastolic blood pressure (−7.69%, p < 0.001; and −9.47%, p < 0.001), serum triglyceride levels (−34.57%, p < 0.001), total cholesterol (−8.70%, p < 0.001), LDL cholesterol (−13.69%, p < 0.001), HbA1c (−3.70%, relative value, p < 0.001), fasting blood glucose (−6.10%, p < 0.001), fasting insulin (−40.86%, p < 0.001) and HOMA-I (−45.71%, p < 0.001), whereas HDL cholesterol increased (14.85%, p < 0.001). None of these parameters changed in participants of the control arm (Table 1). The observed changes of these clinical and laboratory parameters are in agreement with previous studies evaluating the efficacy of the ABC program in clinically distinct cohorts^17^,^18^.

### BA profile

To assess the influence of lifestyle-induced weight loss on serum BAs, BA profiling was performed at baseline and after 6 months in both study arms. In order to avoid excessive multiple testing the 15 analysed BA species were grouped according to Table 2.

We observed a significant decrease of total BA levels in participants of the ABC arm (−34.59%, p = 0.007) that resulted mainly from a decrease of glycine and taurine conjugated BAs (−49.11%, p = 0.002 and −54.46%, p = 0.001) (Fig. 1b and Supplementary Table S2). Regarding different BA-fractions, lifestyle-induced weight loss was accompanied by a significant decrease of total CDCA (−52.38%, p = 0.001), CA (−31.49%, p = 0.018) and UDCA (−49.37%, p = 0.002) (Fig. 1c and Supplementary Table S2). The reduction of total CDCA and CA was caused by a decrease of the glycine or taurine conjugated BAs and the reduction of total UDCA was caused by a decrease of its unconjugated or glycine conjugated form (Supplementary Table S2). No significant alterations of the BA profile were observed in participants of the control arm. Taurine conjugated UDCA and taurine conjugated LCA were below the limit of detection in participants of both arms. As total LCA and DCA did not change, these BA groups were excluded from further analysis.

To determine whether the improvement of insulin sensitivity following lifestyle-induced weight loss was associated with a reduction of the 12α-hydroxylated/ non-12α-hydroxylated ratio we next analysed these two BA subcategories in both arms. Contradictory to our hypothesis the decrease of non-12α-hydroxylated BAs was more pronounced than that of 12α-hydroxylated BAs, resulting in a significantly increased 12α-hydroxylated/ non-12α-hydroxylated ratio (p = 0.001) following lifestyle-induced weight loss (Fig. 1d, Supplementary Table S2). The detailed characterisation of the BA pool composition did not identify any BA species that increased during lifestyle-induced weight loss.

### Fasting FGF-19 and total GLP-1 plasma levels

**Figure 2:**
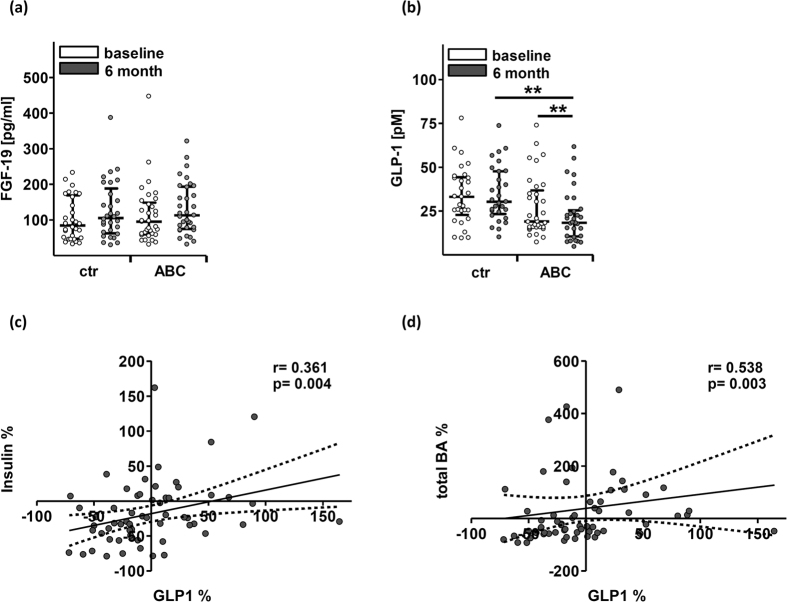
Changes of fibroblast growth factor 19 (FGF-19) and glucagon-like peptide-1 (GLP-1) in subjects with MetS who underwent lifestyle-induced weight loss (ABC) and in controls. Concentrations of FGF-19 (**a**) and GLP-1 (**b**) are shown as individual data points and median ± interquartile range. Wilcoxon Signed-Rank Test was used to analyse differences between paired samples; **p < 0.01. Spearman correlation (2-tailed) was used to analyse correlation between relative changes of insulin (**c**) and GLP-1 or total bile acids (BA) and GLP-1 (**d**) respectively. Data are shown as individual data points. Regression line is given as mean ± 95% confidence interval.

Lifestyle-induced weight loss had no effect on circulating FGF-19 levels (Fig. 2a), while total GLP-1 decreased significantly (p = 0.003; Fig. 2b). Both parameters remained stable in the control arm. Hence, decrease of total GLP-1 may be attributed to lifestyle-induced weight loss and the associated metabolic improvements. Accordingly, relative changes of GLP-1 correlated significantly with relative changes of insulin, individual BAs and other metabolic parameters related to the MetS (Fig. 2c,d; Supplementary Table S3). Together, current results indicate an association between weight loss, serum BAs, insulin and GLP-1.

### Bivariate correlations between metabolic markers, BAs and GLP-1 during lifestyle-induced weight loss

We next determined bivariate correlations of relative changes of grouped BA fractions and GLP-1 between baseline and 6 month with relative changes of body weight and metabolic parameters that may influence BA metabolism, insulin, triglycerides and total cholesterol (Supplementary Table S3).

We observed moderate to weak correlations between total BAs and GLP-1 (r = 0.54; p < 0.001), insulin (r = 0.41; p < 0.001), triglycerides (r = 0.36; p = 0.004), cholesterol (r = 0.32; p = 0.011), and body weight (r = 0.28; p = 0.028).

Interestingly, changes of fasting insulin correlated with all analysed BA groups (Supplementary Table S3). Moderate correlations were observed between insulin and changes of total CDCA (r = 0.45; p < 0.001), total UDCA (r = 0.47; p < 0.001) and non-12α-hydroxylated BAs (r = 0.44; p < 0.001).

Total GLP-1 correlated moderately or weakly with relative changes of all analysed BA groups. Moderate to weak correlations were also found between GLP-1 and cholesterol (r = 0.42; p = 0.001), body weight (r = 0.39; p = 0.002), and insulin (r = 0.36; p = 0.005).

Taken together, our data support the existence of a feedback loop between BA, triglyceride and cholesterol metabolism that is associated with insulin and GLP-1.

### Multiple regression analysis between BAs, GLP-1 and parameters of the metabolic syndrome

Because total BAs, individual BA subgroups and GLP-1 were correlated with relative body weight change, insulin, triglycerides and cholesterol, we performed a linear regression analysis to identify which of these variables are predictive for changes of BAs and GLP-1 by backward elimination. Considering relative changes of body weight, insulin, triglycerides and cholesterol as independent determinant variables, we observed associations between body weight and total CDCA (β = 0.28; p = 0.030) and non-12α-hydroxylated BAs (β = 0.28; p = 0.028), between insulin and total UDCA (β = 0.36; p = 0.004) and total taurine conjugated BAs (β = 0.34; p = 0.008), between triglycerides and total secondary BAs (β = 0.25; p = 0.030) and between cholesterol and total GLP-1 (β = 0.34; p = 0.009).

None of these BA groups were determined by more than one of the selected variables and the determinant variables differed for each group. We have not identified associations between BA groups and changes of cholesterol, indicating that cholesterol levels do not predict serum BA pool composition. In summary, our data indicate that relative changes of body weight, insulin, triglycerides and cholesterol are not predictive for BA changes in a common model.

## Discussion

We analysed the relationship between individual endogenous BA levels and metabolic parameters following lifestyle-induced weight loss in individuals with MetS. Remarkably, weight loss was accompanied by a significant reduction of serum BA concentrations and GLP-1 levels. Lifestyle-induced changes of BAs and GLP-1 were correlated with changed levels of insulin, cholesterol, and triglycerides. FGF-19 levels did not change following lifestyle-induced weight loss, indicating that intestinal FGF-19 and GLP-1 secretion might not be coupled. Detailed analysis of BA species showed that no individual BA increased. Multiple regression analysis revealed that clinical and metabolic parameters, i.e. body weight, plasma insulin, triglycerides and cholesterol, are not suitable to predict serum BA composition by a common model.

Our data are in contrast to observations made in patients who underwent gastric bypass surgery, which is characterised by an increase of BAs, FGF-19 and GLP-1 concentrations^9^,^12^,^13^,^14^. BA increase following gastric bypass surgery has been shown to be biphasic, with an early increase (one month post-surgery) of UDCA and its conjugates and a gradual increase within 24 months post-surgery of CDCA, CA, DCA and GDCA^19^. Hence, it is discussed that the increase of BA species may promote early improvement of insulin sensitivity following gastric bypass surgery independently from weight loss. However, other bariatric surgery procedures that preserve integrity of the intestine, like laparoscopic sleeve gastrectomy or gastric banding, are not associated with elevated BA levels, despite improved insulin sensitivity^16^,^20^,^21^,^22^. Taken together, surgical manipulations of the intestine increase or at least prevent a weight loss associated decrease of BAs, as observed in the current study. It is possible that bariatric surgery procedures are accompanied by an altered enterohepatic recirculation of BAs^9^,^13^, which counteracts a weight loss associated decrease of serum BA levels.

Despite different post-surgery BA-responses the weight-adjusted long-term improvement of insulin sensitivity is comparable following laparoscopic sleeve gastrectomy, gastric banding, or bariatric bypass surgery^23^. Indeed, some authors argue that reduced caloric intake and weight loss, but not increased serum BA levels, improve glucose metabolism following bariatric surgery^10^,^16^. The current results, demonstrating that serum BA levels even decrease following lifestyle-induced weight loss, support the argument that reduced caloric intake and weight loss, and not increased serum BA levels, improve insulin sensitivity.

Since lifestyle-induced weight loss is accompanied by reduced serum BA concentrations, it might be possible that attenuated activation of FXR promotes the development of hepatic steatosis^24^. Hepatic steatosis is strongly associated with insulin resistance and various features of the MetS. Insulin stimulates SREBP1c mRNA transcription and posttranslational proteolytic activation, leading to an activation of hepatic lipogenic gene expression^25^. However, our results demonstrate that lifestyle-induced weight loss, which leads to improved insulin sensitivity, is associated with reduced serum TG, ALAT and ASAT levels. Thus, our results indicate that reduced serum BA levels following lifestyle-induced weight loss in individuals with MetS are not associated with hepatic steatosis.

Our data raise the question as to why BA levels that were reported to be increased in individuals with MetS^15^,^26^,^27^ decrease following weight-loss associated improvement of insulin sensitivity. 95% of the BAs are reabsorbed at the distal ileum and transported back to the liver via portal circulation, where >90% of reabsorbed BAs are efficiently extracted. It has been shown that primary BA pool size represents a major determinant of serum BA levels following cholecystokinin treatment^28^. Based on this, it is suggested that serum BA concentrations reflect spillover of BAs into systemic circulation. In healthy individuals, hepatic BA synthesis is inhibited by BAs itself via activation of FXR, with CDCA being the most efficacious endogenous FXR ligand^29^. Activation of FXR inhibits transcription of the critical regulatory gene in BA synthesis, cholesterol 7alpha-hydroxylase (CYP7A1). Several observations indicate that this feedback mechanism is impaired in diabetes^30^,^31^,^32^. Accordingly, it was shown that FXR gene expression is controlled by insulin and reduced under diabetic conditions^31^. Our results demonstrate that the decrease of serum BA levels correlated with reduced fasting insulin levels. Hence, we assume that improved insulin sensitivity following lifestyle-induced weight loss restored physiologic control of FXR by BAs, resulting in reduced serum BA levels. Our data suggest that reduced BA levels are a consequence and not a cause of improved metabolic control during lifestyle-induced weight loss.

Different BA species have distinct selectivity for BA receptors. Thus, alterations of serum BA composition may interfere with metabolic functions. To our knowledge, this is the first study describing alterations of BA composition following life style induced weight loss in human individuals. The decrease of BAs was mainly restricted to CDCA (non-12α-hydroxylated primary BA), its secondary metabolite UDCA, and to a lesser extent to CA (12α-hydroxylated primary BA). Weight loss resulted in an increased ratio of 12α-hydroxylated/ non-12α-hydroxylated BAs. The ratio of CA (12α-hydroxylated) over CDCA (non-12α-hydroxylated) is controlled by hepatic CYP8B1, which is responsible for the synthesis of CA and its derivatives^33^. The 12α-hydroxylated BAs CA and DCA are reported to be elevated in insulin resistant individuals^5^,^15^,^34^ and genetic deletion of CYP8B1 in mice leads to improved glucose tolerance^35^. Based on those observations our results may appear unexpected. However, an increased ratio of 12α-hydroxylated/ non-12α-hydroxylated BAs was also observed 2 years after bariatric surgery^36^, suggesting a common mechanism that might be mediated by improved hepatic insulin sensitivity.

Another potential factor, which may contribute to the decreased serum BA concentrations, is an altered first pass extraction by the liver. Furthermore, BA pool composition depends on the gut microbiome, which might be changed following lifestyle-induced weight loss. Bacteria convey intestinal deconjugation and dihydroxylation of primary BAs, which gives rise to the secondary BAs DCA, LCA and UDCA^30^,^37^. The possible relevance of the gut microbiome for the observed alterations of serum BA composition was not addressed in the current study and awaits further investigations.

Enhanced faecal elimination of BAs by a fibre rich diet, as recommended to the study participants, is another potential cause for the decreased serum BA concentrations. The dietary intake was not monitored in the current study and therefore BA reduction cannot be correlated to the actually fibre intake. However, faecal BA loss is typically compensated by an enhanced hepatic LDL uptake and by an enhanced synthesis of BAs. FGF-19 acts as a negative feedback regulator of hepatic BA synthesis^38^, and increased intestinal BA elimination by sequestrants or a fibre rich diet results in decreased intestinal FGF-19 secretion and lower FGF-19 plasma levels^39^. Yet we observed no change of FGF-19 levels following weight loss, arguing against faecal BA elimination as a major factor contributing to the observed BA reduction.

Glucose homeostasis is thought to be improved by BAs through induction of intestinal GLP-1 secretion via activation of TGR5^6^. In concordance with that, gastric bypass surgery is associated with increased plasma levels of GLP-1^12^, even in the fasting state^14^. GLP-1 is an incretin and a potent stimulator of insulin secretion from pancreatic β-cells, thus promoting postprandial glucose clearance. Furthermore, GLP-1 inhibits gastric emptying and promotes satiety^40^,^41^.

Unlike gastric bypass surgery lifestyle-induced weight loss was not associated with elevated GLP-1 levels in our study. Instead, we observed decreased levels of fasting GLP-1. The decreased levels of fasting GLP-1 were correlated with changes of insulin and total BA levels. Furthermore, multiple regression analysis revealed an association between cholesterol and GLP-1. It has been shown that elevated fasting levels of GLP-1 are associated with MetS components^42^ and that decreased fasting GLP-1 levels correlate with fasting insulin levels in obese individuals who reduced body weight by dietary changes^43^. Based on these studies, we assume that the observed reduction of GLP-1 may reflect recovered metabolic homeostasis following lifestyle-induced weight loss.

Strengths of the current study include the prospective study design, a well characterised study population with no differences between groups at baseline, a low dropout rate and high compliance of participants as a result of daily telemonitoring and weekly letters commenting individual weight progress. Laboratory technicians were blinded regarding to the status of the samples and all laboratory measurements were performed according to standard operating protocols. As a result, obtained data are of high quality. On the other hand, the current study is limited to middle aged male caucasian participants with MetS. Thus the results cannot be generalised to other racial/ethnic groups and to individuals without MetS. Weight loss in the present study was less pronounced compared to bariatric surgery and the observational period was limited to 6 months. Hence, our study does not address longitudinal changes in serum BA levels following endogenous weight loss. Another limitation is that only fasting levels were measured. Therefore, we cannot comment on postprandial changes of serum BAs. The impact of an endogenous weight loss on postprandial serum BA levels remains to be evaluated in future studies. Furthermore, total GLP-1 was estimated instead of active GLP-1. Active GLP-1 has a very short half-life and is found in low concentrations, before it is degraded by DPP-IV, while total GLP-1 gives an indication of the secretion from intestinal L-cells. However, total GLP-1 has been shown to positively correlate with active GLP-1 concentration^44^.

Taken together, using a telemonitored program we achieved a pronounced weight loss and metabolic improvement in obese, pre-diabetic individuals. Importantly, in contrast to bariatric surgery lifestyle-induced weight loss results in a decrease of serum BA and GLP-1 concentrations without affecting FGF-19. These changes are associated with specific alterations of serum BA composition resulting in an increased 12α-hydroxylated/non-12α-hydroxylated ratio. Therefore, lifestyle-induced metabolic changes cannot be explained by increased serum levels of BAs, GLP-1, or FGF-19. Current results suggest instead that lower levels of BAs and GLP-1 are the consequence of improved insulin sensitivity and the associated recovered metabolic homeostasis. These results provide important novel insights regarding the role of BAs in glucose homeostasis and their therapeutic potential for insulin resistance.

## Materials and Methods

### Research design

The study is embedded in a prospective, two-armed, controlled, mono-centric, randomised, 6-months intervention trial to identify changes of the mRNA and microRNA expression profile of individuals with metabolic syndrome following lifestyle-induced weight loss. For this purpose paired blood samples and subcutaneous adipose tissue biopsies were collected at the Institute of Clinical Chemistry and Pathobiochemistry, Otto-von-Guericke University, Magdeburg, Germany. The trial was registered at the German Clinical Trials Register (ICTRP Trial Number: U1111-1158-3672) in July 2014.

The trial included non-smoking, non-diabetic men aged between 45 and 55 years with MetS as defined by the National Cholesterol Education Program Adult Treatmen Panel III guideliens: abdominal obesity (waist circumference > 102 cm or BMI > 30 kg/m^2^) combined with at least two of the following criteria: fasting triglyceride concentration ≥ 1.7 mmol/l; high-density lipoprotein (HDL) cholesterol < 1.05 mmol/l; fasting glucose ≥ 5.6 mmol/l; blood pressure ≥ 130/85 mmHg or treatment for hypertension. Exclusion criteria were smoking, diabetes mellitus type 2, surgical procedure for weight loss within the previous 6 months, severe renal dysfunction (creatinin concentration > 2.0 mg/dl), active liver disease, obesity of known endocrine origin or inability to walk at least 30 minutes per day. Participants were recruited by an advertisement in a regional newspaper. Out of 133 individuals screened for inclusion or exclusion criteria from May 2012 to August 2012, 74 individuals were selected for the trial. All participants underwent a structured education about diet and the importance of physical activity. Individuals were randomly assigned to a 6 months lasting telemonitored lifestyle-induced weight loss program (ABC) or a control arm by a web-based randomisation tool using permuted block randomisation with stratification on BMI (Randomisation In Treatment Arms (RITA); University of Lübeck, Germany). A blinded investigator who did not have any interaction with the participants during the screening and enrolment process managed group allocation assignments. Participants of both arms were linked to an identification number and samples were collected in sequentially numbered containers. Hence, laboratory technicians were blinded regarding to the status of the samples. Participants of the ABC arm received accelerometers measuring their daily activity and instructions for daily data transmission of body weight, physical activity and approximated caloric intake^17^. Participants of the ABC arm received regular feedback by weekly letters commenting their individual weight progress and daily exercise-related energy expenditure. At baseline and after 6 months subcutaneous adipose tissue biopsies and peripheral blood samples were obtained from participants of both arms. All study participants were examined after 3 months and screened for fasting blood glucose and glycated haemoglobin (HbA1c) to prevent complications secondary to progression to overt type 2 diabetes. Expression profile of microRNAs in paired blood and subcutaneous adipose tissue will be analysed as primary outcome (not part of this report). Changes of bile acid concentrations were quantified as secondary outcome at baseline and at the end of the study period in paired serum samples.

### Ethics Statement

This study was approved by the ethics committee at Otto-von-Guericke University, Magdeburg, Germany (No. 78/11). Written informed consent was obtained from all study participants. All human investigations were conducted according to the principles expressed in the Declaration of Helsinki.

### Clinical and laboratory parameters

Body weight, height, waist circumference and blood pressure were measured by qualified medical personnel according to standard operating protocols at baseline and after 6 months. Body composition was analysed by dual-energy X-ray absorptiometry.

All blood samples were collected in the morning (8 am to 9 am) from the antecubital vein after a 12-hour overnight fast. BA profiling was performed by high performance liquid chromatography-tandem mass spectrometry at the Department of Clinical Chemistry, Karolinska University Hospital Huddinge, Stockholm, Sweden. Deuterium labelled unconjugated and glycine or taurine conjugated BAs were used as internal standards for quantification of the primary and secondary BAs CA, CDCA, DCA, LCA and UDCA^45^.

All other laboratory measurements were performed at the Institute of Clinical Chemistry and Pathobiochemistry, OvGU, Magdeburg, Germany. Fasting blood glucose, triglycerides, ALAT, ASAT, low-density lipoprotein (LDL) cholesterol, HDL cholesterol and total cholesterol were analysed by commercial enzymatic methods using a random-access analyser (Modular, Roche Diagnostics, Mannheim, Germany). Lipoprotein fractions were analysed by ultracentrifugation. Glucose was determined in sodium fluoride plasma. HbA1c was determined by high performance liquid chromatography. Insulin was determined by ^125^I-radioimmunoassay (RIA) according to the manufacturer’s instructions (INSULIN-CT, CIS bio, Berlin, Germany).

The homeostasis model of assessment index (HOMA-I) was calculated using fasting insulin and glucose values^46^. Plasma FGF-19 concentrations were determined by a commercial ELISA assay (FGF-19 Quantikine ELISA, R&D Systems, Minneapolis, Minnesota, USA). Total plasma GLP-1 was analysed using a RIA with antibodies targeting the C-terminal of GLP-1 after ethanol extraction (GLP1T-36HK, Linco Research, St. Charles, Missouri, USA), according to manufacturer’s instructions.

### Statistical analysis

Sample size was determined to characterize the expression profile of microRNAs in paired blood and subcutaneous adipose tissue from individuals with MetS following lifestyle-induced weight loss. The following sample size computational model was used:

http://bioinformatics.mdanderson.org/MicroarraySampleSize/MicroarraySampleSize.aspx. We aimed to target the upper 75th percentile as level of variance across all genes and applied a level of 0.65 as a realistic value for standard deviation of the gene intensity measurement on the base-2 logarithmic scale for genes that are expressed at moderate to high levels^47^. This calculation resulted in a sample size of n = 29 required for detecting a 1.5-fold change in 75% least variable genes with a two sided 0.001 significance level (false positive rate of 1%) and power of 80%. With a supposed dropout rate of 20% 37 individuals had to be recruited for each group.

Changes of bile acid concentrations were quantified as secondary outcome. As data of BA-profiling were not normally distributed, non-parametric tests were applied for all statistical analysis. Data are given as median and interquartile range (IQR). Differences between independent samples (ABC vs. control at baseline or 6 month, respectively) were analysed by Mann-Whitney U Test. Paired samples were analysed by Wilcoxon Signed-Rank Test. Correlations between relative changes of BAs and other variables were assessed by Spearman’s rank correlation. Multiple linear regression analysis with backward elimination was used to identify which of the determinant variables that were significantly changed after weight loss and may influence BA metabolism (body weight, insulin, cholesterol, triglycerides) were independently associated with altered BA groups or GLP-1. All calculations were performed using the IBM® SPSS® Statistics, version 22.0 (IBM Corporation, Armonk, NY, USA). Results were considered significant at *P* < 0.05.

## Additional Information

**How to cite this article**: Biemann, R. *et al*. Serum bile acids and GLP-1 decrease following telemetric induced weight loss: results of a randomized controlled trial. *Sci. Rep.* **6**, 30173; doi: 10.1038/srep30173 (2016).

## Supplementary Material

Supplementary Information

## Figures and Tables

**Table 1:** Clinical characteristics of subjects with MetS who underwent lifestyle-induced weight loss (ABC) and controls at baseline and after 6 months.

		ABC		Control	
		Baseline	6 month	Baseline	6 month
age	median	48		48	
	IQR	(45.0–51.50)		(43.75–51.25)	
BMI	median	33,02	28.95***	33.41	33.57
	IQR	(31.16–35.75)	(27.31–31.40)	(31.7–37.07)	(32.36–35.78)
body weight [kg]	median	111.0	95.0***	105.0	107.25
	IQR	(100.0–118.0)	(85.45–103.15)	(99.3–122)	(99.5–122.25)
RRsys [mmHg]	median	142.0	130.0***	140.0	140.0
	IQR	(130.0–151.0)	(120.0–135.0)	(125–158.5)	(130–140.5)
RRdia [mmHg]	median	90.0	82.0***	90.0	90.0
	IQR	(82.0–99.0)	(80.0–86.0)	(83.5–97.0)	(80.0–93.0)
triglycerides [mmol/l]	median	2.06	1.37***	2.0	2.04
	IQR	(1.38–3.94)	(1.09–1.99)	(1.51–2.62)	(1.54–3.55)
cholesterin [mmol/l]	median	6.16	5.69***	5.90	5.78
	IQR	(5.32–6.91)	(4.50–6.31)	(5.19–6.91)	(4.99–7.07)
HDL-cholesterin [mmol/l]	median	1.20	1.41***	1.33	1.37
	IQR	(1.02–1.40)	(1.19–1.58)	(1.11–1.53)	(1.11–1.51)
LDL-cholesterin [mmol/l]	median	3.74	3.35***	3.44	3.24
	IQR	(3.08–4.70)	(2.37–4.11)	(2.95–4.39)	(2.65–4.14)
fasting insulin [pmol/l]	median	88.0	47.0***	62.5	61.0
	IQR	(59.0–134.0)	(25.0–70.0)	(50.0–88.5)	(47.0–90.25)
fasting glucose [mmol/l]	median	6, 12	5.53***	5.95	5.93
	IQR	(5.61–6.31)	(5.25–5.97)	(5.55–6.27)	(5.43–6.36)
HOMA	median	3.15	1.71***	2.40	2.20
	IQR	(2.19–5.01)	(0.94–2.56)	(1.89–3.31)	(1.66–3.78)
HbA1c [%]	median	5.60	5.40 ***	5.60	5.60
	IQR	(5.30–6.05)	(5.20–5.60)	(5.37–5.85)	(5.30–5.80)
HbA1c [mmol/mol]	median	37.0	35.0***	38.0	37.0
	IQR	(34.0–43.0)	(33.0–37.0)	(35.5–40.5)	(34.75–40.0)
fat mass [kg]	median	31.69	22.98***	28.23	30.02
	IQR	(25.86–36.31)	(20.30–28.46)	(25.43–40.6)	(25.44–40.77)
trunk fat mass [kg]	median	17.91	13.38***	17.43	18.14
	IQR	(15.24–21.80)	(10.39–16.32)	(14.15–23.48)	(14.27–23.15)
fat free mass [kg]	median	75.55	70.36***	72.91	73.46
	IQR	(67.69–78.69)	(65.25–74.88)	(68.65–79.28)	(69.39–79.87)
ALAT [μmol/s·l]	median	0.64	0.40***	0.74	0.685
	IQR	(0.515–0.975)	(0.320–0.535)	(0.46–1.07)	(0532–1.052)
ASAT [μmol/s·l]	median	0.48	0.41***	0.500	0.530
	IQR	(0.43–0.65)	(0.34–0.44)	(0.395–0.615)	(0.407–0.632)

Data are presented as median (interquartile range). Wilcoxon Signed-Rank Test was used to analyse differences of paired samples with N = 33 ABC, N = 30 control, ***p < 0.01. No differences were found between ABC vs. control at baseline, analysed by Mann-Whitney U Test.

**Table 2:**
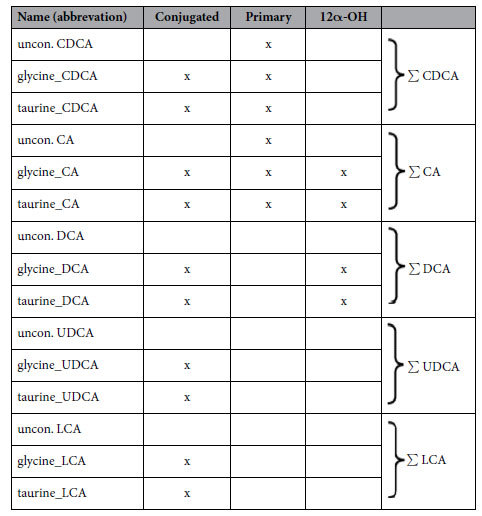
Subgrouping of the measured bile acids.

CDCA, chenodeoxycholic acid; CA, cholic acid; DCA, deoxycholic acid; UDCA, ursodeoxycholic acid; LCA, lithocholic acid; 12α-OH, 12α-hydroxylated BAs.
