# Supplementary material for: Serum bile acids and GLP-1 decrease following telemetric induced weight loss: results of a randomized controlled trial
Source: Sci Rep. 2016 Jul 25;6:30173. doi: 10.1038/srep30173 (PMC4958948; doi:10.1038/srep30173)
Supplement: Supplementary Information [file srep30173-s1.pdf]

Supplementary Information

Serum bile acids and GLP-1 decrease following telemetric induced weight loss: results of a randomized controlled trial

Ronald Biemann[1], Marina Penner[1], Katrin Borucki[1], Sabine Westphal[1], Claus Luley[1], Raik Rönicke[1], Kathleen Biemann[1], Cornelia Weikert[2,3], Anke Lux[4], Nikolai Goncharenko[5], Hanns-Ulrich Marschall[6], Jochen G. Schneider[7,8]* and Berend Isermann[1]*

Supplementary Table S1

Absolute and relative weight reduction in completers and after inclusion of dropout cases according to the LOCF and BOCF evaluation procedures.

		ABC			control			control vs. ABC
		n	median	IQR	n	median	IQR	*p*
absolute weight loss [kg]	completers	33	-13.2	-18 to -11	30	0.9	-2 to 2	**0.000**
	LOCF	35	-13.1	-18 to -9	37	0.9	-2 to 2	**0.000**
	BOCF	37	-12.7	-18 to -8	37	0.0	-1 to 2	**0.000**
relative weight loss [%]	completers	33	-12.6	-16 to -10	30	0.9	-1 to 2	**0.000**
	LOCF	35	-12.6	-16 to -8	37	0.9	-2 to 2	**0.000**
	BOCF	37	-12.3	-16 to -8	37	0.0	-1 to 2	**0.000**

Mann-Whitney U Test was used to analyze differences between groups. LOCF, last observation carried forward; BOCF, baseline observation carried forward.

Supplementary Table S2

Bile acid (BA) concentrations in subjects with metabolic syndrome who underwent lifestyle-induced weight loss (ABC) and controls at baseline and after 6 months.

bile acid fractions [μM]	Wilcoxon Signed-Rank Test				Mann-Whitney-U Test	
	ABC		control		control vs. ABC	
	baseline	6 month	baseline	6 month	baseline	6 month
∑ CDCA	1.084 (0.579-2.125)	0.513*** (0.192-0.830)	1.033 (0.426-1.576)	0.831 (0.486-1.446)	n.s.	*
uncon. CDCA	0.261 (0.115-0.704)	0.146* (0.043-0.413)	0.297 (0.143-0.679)	0.189 (0.122-0.465)	n.s.	n.s.
glycine_CDCA	0.548 (0.242-0.984)	0.268*** (0.127-0.423)	0.614 (0.280-0.920)	0.450 (0.233-0.811)	n.s.	**
taurine_CDCA	0.060 (0.028-0.091)	0.028*** (0.015-0.040)	0.039 (0.027-0.091)	0.059 (0.023-0.084)	n.s.	*
∑ CA	0.408 (0.245-0.806)	0.287* (0.166-0.510)	0.353 (0.241-0.762)	0.318 (0.219-0.703)	n.s.	n.s.
uncon. CA	0.136 (0.106-0.407)	0.141 (0.084-0.304)	0.134 (0.086-0.269)	0.124 (0.094-0.285)	n.s.	n.s.
glycine_CA	0.189 (0.097-0.310)	0.119** (0.066-0.193)	0.179 (0.110-0.262)	0.158 (0.124-0.233)	n.s.	*
taurine_CA	0.019 (0.009-0.039)	0,009*** (0.000-0.019)	0.024 (0.008-0.043)	0.020 (0.009-0.036)	n.s.	*
∑ DCA	0.905 (0.602-1.244)	0.666 (0.405-1.161)	0.825 (0.380-1.197)	0.665 (0.345-1.077)	n.s.	n.s.
uncon. DCA	0.460 (0.327-0.835)	0.439 (0.267-0.771)	0.413 (0.187-0.763)	0.416 (0.217-0.640)	n.s.	n.s.
glycine_DCA	0.269 (0.155-0.542)	0.200* (0.070-0.365)	0.231 (0.090-0.489)	0.201 (0.105-0.432)	n.s.	n.s.
taurine_DCA	0.031 (0.016-0.051)	0.018* (0.009-0.036)	0.019 (0.008-0.034)	0.022 (0.008-0.035)	n.s.	n.s.
∑ UDCA	0.214 (0.100-0.368)	0.098** (0.041-0.207)	0.162 (0.065-0.330)	0.157 (0.067-0.262)	n.s.	n.s.
uncon UDCA	0.077 (0.037-0.196)	0.047* (0.006-0.120)	0.065 (0.000-0.176)	0.071 (0.017-0.133)	n.s.	n.s.
glycine_UDCA	0.880 (0.034-0.179)	0.045** (0.022-0.094)	0.088 (0.036-0.141)	0.076 (0.030-0.133)	n.s.	n.s.
taurine_UDCA	0.00 (0.000-0.000)	0.00 (0.000-0.000)	0.000 (0.000-0.000)	0.000 (0.000-0.000)	n.s.	n.s.
∑ LCA	0.069 (0.044-0.094)	0.056 (0.043-0.084)	0.062 (0.042-0.094)	0.062 (0.044-0.077)	n.s.	n.s.
uncon. LCA	0.034 (0.010-0.051)	0.031 (0.019-0.049)	0.031 (0.019-0.048)	0.031 (0.020-0.040)	n.s.	n.s.
glycine_LCA	0.030 (0.024-0.044)	0.028* (0.022-0.036)	0.033 (0.024-0.042)	0.030 (0.022-0.039)	n.s.	n.s.
taurine_LCA	0.00 (0.000-0.000)	0.00 (0.000-0.000)	0.000 (0.000-0.000)	0.000 (0.000-0.000)	n.s.	n.s.
∑ Bile Acids	2.837 (1.669-4.273)	1.826** (0.966-2.956)	2.747 (1.505-3.794)	2.116 (1.355-3.433)	n.s.	n.s.
∑ uncon. BA	1.000 (0.707-2.295)	0.883 (0.478-1.682)	1.059 (0.628-1.694)	0.858 (0.563-1.586)	n.s.	n.s.
∑ glycine_BA	1.173 (0.620-2.057)	0.694** (0.362-0.997)	1.318 (0.561-1.882)	0.951 (0.624-1.467)	n.s.	*
∑ taurine_BA	0.123 (0.058-0.157)	0.052*** (0.033-0.075)	0.085 (0.044-0.179)	0.112 (0.037-0.153)	n.s.	*
uncon. BA/con. BA	0.950 (0.446-1.934)	1.143 (0.794-2.542)	0.923 (0.447-1.670)	0.780 (0.517-1.917)	n.s.	n.s.
∑ con. BA	1.306 (0.722-2.257)	0.792*** (0.391-1.091)	1.374 (0.634-2.067)	1.021 (0.642-1.562)	n.s.	*
12α-OH/non-12α-OH	0.975 (0.756-1.318)	1.312*** (0.939-2.090)	0.821 (0.567-1.205)	0.876 (0.591-1.356)	n.s.	**
∑ 12α-OH BA	1.400 (0.908-2.095)	1.003 (0.673-1.660)	1.446 (0.915-1.820)	1.252 (0.725-2.009)	n.s.	n.s.
∑ non-12α-OH BA	1.821 (0.830-2.554)	0.679*** (0.409-1.613)	1.509 (0.790-2.203)	1.266 (0.782-1.866)	n.s.	*
primary/secondary	1.185 (0.827-1.779)	0.957* (0.602-1.653)	1.568 (0.815-2.429)	1.325 (0.888-2.245)	n.s.	*
∑ primary BA	1.584 (0.768-2.961)	0.856** (0.461-1.500)	1.374 (0.687-2.620)	1.162 (0.773-2.028)	n.s.	n.s.
∑ secondary BA	1.273 (0.810-1.638)	0.854* (0.534-1.466)	1.158 (0.675-1.606)	0.934 (0.597-1.291)	n.s.	n.s.

Data are presented as median and interquartile range. Statistically significant correlations are printed bold-faced. Wilcoxon Signed-Rank Test was used to analyze differences of paired samples. Mann-Whitney U Test was used for differences between independent samples; N = 33 ABC, N = 30 control, * $p < 0.05$; ** $p < 0.01$; *** $p < 0.001$. CDCA, chenodeoxycholic acid; CA, cholic acid; DCA, deoxycholic acid; UDCA, ursodeoxycholic acid; LCA, lithocholic acid.

Supplementary Table S3

Spearman-Rho correlation matrix (2-tailed) of relative changes of bile acids and clinical parameters.

body weight															
insulin	.669**														
triglyceride	.577**	.494**													
cholesterol	.409**	.308*	.438**												
GLP-1	.395**	.362**	.271*	.421**											
∑ total BAs	.280*	.414**	.358**	.321*	.539**										
∑ CDCA	.342**	.449**	.402**	.317*	.575**	.967**									
∑ CA	.182	.290*	.305*	.328**	.498**	.891**	.879**								
∑ UDCA	.401**	.472**	.311*	.199	.398**	.766**	.774**	.581**							
∑ con. BA	.314*	.394**	.245	.231	.499**	.815**	.841**	.652**	.722**						
∑ glycine_BA	.313*	.400**	.251*	.219	.487**	.818**	.842**	.652**	.728**	.997**					
∑ taurine_BA	.249	.348**	.229	.301*	.541**	.680**	.702**	.582**	.524**	.826**	.800**				
∑ primary BA	.296*	.396**	.377**	.319*	.560**	.966**	.986**	.935**	.739**	.798**	.802**	.664**			
∑ secondary BA	.231	.359**	.324**	.345**	.477**	.933**	.842**	.756**	.716**	.765**	.759**	.667**	.832**		
∑ non-12α-OH BA	.378**	.441**	.406**	.270*	.506**	.935**	.953**	.792**	.852**	.805**	.807**	.640**	.923**	.855**	
	body weight	insulin	triglyceride	cholesterol	GLP-1	∑ total BA	∑ CDCA	∑ CA	∑ UDCA	∑ con. BA	∑ glycine	∑ taurine	∑ primary	∑ secondary	∑ non-12α-OH

Statistically significant correlations are printed bold-faced; N = 33 ABC, N = 30 control, * $p < 0.05$; ****** $p < 0.01$. BA, bile acids, CDCA, chenodeoxycholic acid; CA, cholic acid; DCA, deoxycholic acid; UDCA, ursodeoxycholic acid; LCA, lithocholic acid..
